# Biofilm-Forming Capacity in Biogenic Amine-Producing Bacteria Isolated from Dairy Products

**DOI:** 10.3389/fmicb.2016.00591

**Published:** 2016-05-12

**Authors:** Maria Diaz, Victor Ladero, Beatriz del Rio, Begoña Redruello, María Fernández, M. Cruz Martin, Miguel A. Alvarez

**Affiliations:** Department of Technology and Biotechnology of Dairy Products, Instituto de Productos Lácteos de Asturias (IPLA-CSIC)Villaviciosa, Spain

**Keywords:** biofilm, biogenic amines, histamine, tyramine, putrescine, lactic acid bacteria, dairy industry

## Abstract

Biofilms on the surface of food industry equipment are reservoirs of potentially food-contaminating bacteria—both spoilage and pathogenic. However, the capacity of biogenic amine (BA)-producers to form biofilms has remained largely unexamined. BAs are low molecular weight, biologically active compounds that in food can reach concentrations high enough to be a toxicological hazard. Fermented foods, especially some types of cheese, accumulate the highest BA concentrations of all. The present work examines the biofilm-forming capacity of 56 BA-producing strains belonging to three genera and 10 species (12 *Enterococcus faecalis*, 6 *Enterococcus faecium*, 6 *Enterococcus durans*, 1 *Enterococcus hirae*, 12 *Lactococcus lactis*, 7 *Lactobacillus vaginalis*, 2 *Lactobacillus curvatus*, 2 *Lactobacillus brevis*, 1 *Lactobacillus reuteri*, and 7 *Lactobacillus parabuchneri*), all isolated from dairy products. Strains of all the tested species - except for *L. vaginalis*—were able to produce biofilms on polystyrene and adhered to stainless steel. However, the biomass produced in biofilms was strain-dependent. These results suggest that biofilms may provide a route via which fermented foods can become contaminated by BA-producing microorganisms.

## Introduction

Food safety is a major priority in today's food industry. Bacterial biofilms on industrial surfaces are a cause for concern since they may act as reservoirs of contaminating microorganisms (Winkelströter et al., 2014). Dairy products in particular are susceptible to such contamination (Srey et al., 2013), with equipment surfaces one of its major sources (Kumar and Anand, 1998). Stainless steel type 304 is the most common material in contact with food in the dairy industry (Zottola and Sasahara, 1994). While inert, easy to clean and highly resistant to corrosion, it can, however, develop small cracks and crevices where biofilm formation is facilitated (Winkelströter et al., 2014). In addition, some parts of food processing equipment may have inaccessible areas where bacteria can evade cleaning treatments. The main biofilm-related risk is the growth of pathogens and spoilage microorganisms.

The capacity of biogenic amine (BA)-producers to form biofilms has not been investigated. BAs are low-molecular weight organic compounds derived from their corresponding amino acids via enzymatic decarboxylation. Although, BAs play an important role in human physiology, the ingestion of food containing them in large quantities can have toxicological effects on the digestive, circulatory, and respiratory systems (ten Brink et al., 1990; Shalaby, 1996; Ladero et al., 2010a). The most important BAs (both qualitatively and quantitatively) in foods and beverages are histamine, tyramine, and putrescine. Together with fish and wine, dairy products—especially cheese—can develop BA concentrations that may exceed 1000 mg kg^−1^ (Linares et al., 2012).

BAs form in food via the activity of bacteria with aminoacyl decarboxylase activity (Halasz et al., 1994). Their appearance in dairy products has mainly been attributed to Gram positive bacteria of the lactic acid bacteria (LAB) group. These can be present in the microbiota of milk, as part of the starter culture, or be introduced by contamination during manufacturing (Linares et al., 2011), with equipment surfaces a potentially important source of contamination (Novella-Rodríguez et al., 2004). The post-ripening processing of cheese, particularly grating, extends the contact of food with equipment surfaces, increasing the number of histamine-producing bacteria present in the final product (Ladero et al., 2009) and therefore the histamine concentration that may be reached.

The dairy histamine-producing species *Lactobacillus parabuchneri* has been reported to produce biofilms (Diaz et al., 2016b), but this capacity has not been studied in other BA producers. The aim of the present work was to test the ability of BA-producing bacteria isolated from different cheeses to form biofilms on polystyrene and adhere to stainless steel surfaces.

## Materials and methods

### Bacterial strains and culture conditions

In this work we have examined the biofilm-forming capacity of 56 BA-producing strains belonging to three genera and 10 species (12 *Enterococcus faecalis*, 6 *Enterococcus faecium*, 6 *Enterococcus durans*, 1 *Enterococcus hirae*, 12 *Lactococcus lactis*, 7 *Lactobacillus vaginalis*, 2 *Lactobacillus curvatus*, 2 *Lactobacillus brevis*, 1 *Lactobacillus reuteri*, and 7 *Lactobacillus parabuchneri*), all isolated from dairy products. The ability of all (except of the *Lactobacillus parabuchneri* strains) to produce BAs was known from previous work (Ladero et al., 2010b,c, 2011a,b, 2012a,b; Diaz et al., 2015; del Rio et al., 2015). Given the reported ability of *L. parabuchneri* to form biofilms (Diaz et al., 2016b), and the apparent importance of the species in the accumulation of histamine in cheese (Diaz et al., 2016a), seven new strains isolated from cheese (following the protocol of Diaz et al., 2015) were included among those examined. These were identified at the species level by *16S rRNA* sequencing (Diaz et al., 2015) and their ability to produce histamine checked by UHPLC (Redruello et al., 2013).

Lactobacilli were grown at 37°C in MRS (Oxoid, Basingstoke, UK), while enterococci and lactococci were grown at 30°C in M17 (Oxoid) supplemented with 0.5% (w/v) glucose (GM17). To test the individual capacity of the *L. parabuchneri* strains to produce histamine, the culture medium was supplemented with 5 mM histidine.

### Analysis of biofilm formation on polystyrene

The ability of the test strains to produce a biofilm on polystyrene was performed as described by Diaz et al. (2016b). Briefly, MRS or GM17 overnight cultures were diluted to approximately 10^6^ cfu mL^−1^ with fresh medium and used to fill polystyrene 96-well microtitre plates (Nunc™ MicroWell™ Plates with a Nunclon™ Delta Surface; Thermo Fisher Scientific, Waltham, MA, USA). Negative controls consisted of wells filled with the corresponding uninoculated culture medium. All plates were then incubated at 30 or 37°C depending on the species. Biofilm biomass was determined using the crystal violet staining method (CV assay) (Kubota et al., 2008). After 24, 30, or 48 h of incubation, the supernatant was removed and the wells rinsed with PBS buffer to eliminate non-adhered cells. The potential biofilm present was then stained with 0.5% (w/v) CV in distilled water (dH_2_O); the excess dye was removed with dH_2_O. The bound dye was then extracted using acetone/ethanol (80:20, v:v) and quantified by absorbance at 595 nm using a Benchmark Plus microplate spectrophotometer (BioRad, Hercules, CA, USA). The mean ± SD of the optical density (OD) of three replicates was calculated for each strain. ANOVA with *post-hoc* Bonferroni correction was used to analyse all data. Significance was set at *p* < 0.05. All statistical calculations were undertaken using SPSS v.15.0 software (SPSS Inc., IL, USA). Biofilm production capacity was expressed using cut-off values (Extremina et al., 2011). The cut-off value between biofilm-producers and non-producers (ODc) was defined as the mean of the negative controls (ODnc) plus three SDs. The strains were then classified as belonging to one of the following categories: ODc < OD ≤ 2 × ODc = weak biofilm producer, 2 × ODc < OD ≤ 4 × ODc = moderate biofilm producer, and OD > 4 × ODc = strong biofilm producer.

### Analysis of bacterial adherence to stainless steel

The test surfaces used were 1 cm^2^ stainless steel (type AISI 304) coupons. These were washed with soap and dH_2_O, rinsed with dH_2_O, and then immersed in acetone for 30 min to remove any grease or fingerprints. They were then rinsed once again in dH_2_O, autoclaved, and immersed singly in tubes containing MRS or GM17 broth inoculated with 10^6^ cfu mL^−1^ of the assayed strain (performed in triplicate). Each coupon was then incubated at 30 or 37°C for 24 or 48 h before removal using sterile forceps. Non-adhered cells were removed by rinsing the coupon three times in PBS buffer. The coupons were then re-immersed in 5 mL PBS buffer, and the adhered cells detached from the coupon by sonication in an ultrasonic bath (Ultrasons-H, Selecta, Spain) for 15 min. The bacterial suspension produced was serially diluted in PBS, and 100 μL of 10^0^, 10^−1^, 10^−2^, 10^−3^, and 10^−4^ dilutions were plated on MRS or GM17 and incubated for 48 h (Kruszewski et al., 2013). Three replicates were performed for each strain using independent bacterial cultures. Bacterial counts were expressed as log_10_ cfu cm^−2^ (mean ± SD of three replicates). To confirm the tolerance of the cells to sonication, bacterial suspensions of all the examined strains were sonicated for 15 min. Pre- and post-sonication suspensions were serially diluted in PBS, plated, incubated for 48 h, and the cells enumerated; no significant differences were seen between pre- and post-sonication cell counts confirming that no cells were killed by this procedure.

### Scanning electron microscopy of cells adhered to stainless steel

The method of Kubota et al. (2008) was followed, with some modifications, to observe by scanning electron microscopy (SEM) the cells adhered to the stainless steel coupons. Briefly, the latter were rinsed twice in PBS and then fixed in 2.5% glutaraldehyde (Sigma-Aldrich) in PBS for 16 h at room temperature. The fixed bacteria were then dehydrated using a graded series of acetone solutions (50–100% v/v), and the coupons dried with CO_2_ using a CPD-030 critical point dryer (Bal-Tec AG, Balzers, Liechtenstein). They were then coated with gold using a SCD 004 Sputtering Coater (Bal-Tec AG, Balzers, Liechtenstein) and observed using a JSM-6610LV SEM (JEOL USA, Inc, Peabody, MA, USA).

## Results

### Biofilm formation on polystyrene

Maximum biofilm biomass values were obtained at different times of incubation depending on the species. For all the *Enterococcus* strains assayed, biofilm biomass was maximal at 30 h; for all the *L. lactis, L. curvatus*, and *L. brevis* strains, maximum values were reached at 24 h; and for the *L. reuteri* and *L. parabuchneri* strains, maxima were recorded at 48 h. All incubation time results shown are those at which maximum biomass was reached.

**Figure 1:**
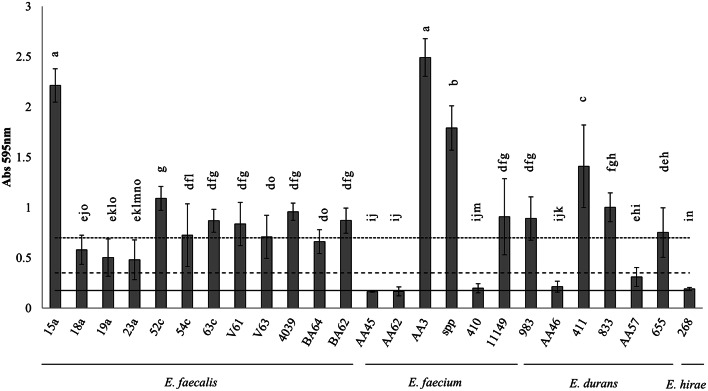
**Biofilm-producing capacity on polystyrene of the biogenic amine-producing *Enterococcus* strains after 30 h of incubation at 30°C**. Data represent means ± SD (error bars) of three experiments. Values marked with the same letter do not differ significantly (*p* > 0.05 according to the Bonferroni *post-hoc* test). (___) Cut-off line (ODc); (__ __ __) 2 × ODc; (…….) 4 × ODc.

All the *E. faecalis* strains were able to form biofilms on polystyrene. Six were classified as strong biofilm producers, and six as moderate biofilm producers (Figure 1). The only *E. hirae* strain assayed was a weak biofilm producer (Figure 1). All the *E. durans* strains tested were able to form biofilms; four were strong biofilm producers, and two were weak producers (Figure 1). Of the six *E. faecium* strains analyzed, three were strong biofilm producers, one was a weak producer, and two were unable to form a biofilm (Figure 1).

**Figure 2:**
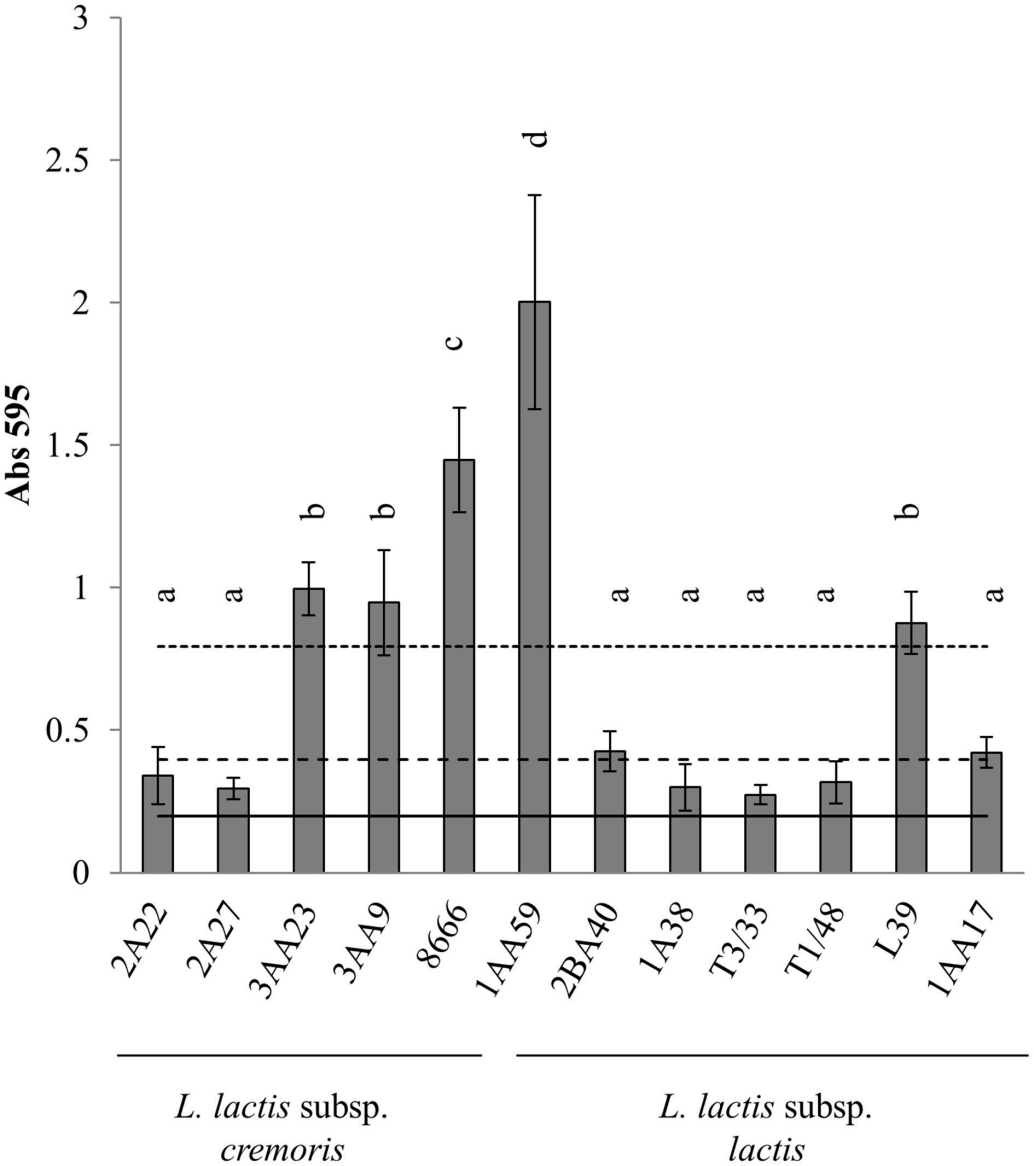
**Biofilm-producing capacity on polystyrene of the biogenic amine-producing *Lactococcus* strains after 24 h of incubation at 30°C**. Data represent means ± SD (error bars) of three experiments. Values marked with the same letter do not differ significantly (*p* > 0.05 according to the Bonferroni *post-hoc* test). (___) Cut-off line (ODc); (__ __ __) 2 × ODc; (…….) 4 × ODc.

All the *L. lactis* strains were able to produce biofilms on polystyrene (Figure 2). Three *L. lactis* subsp. *cremoris* and two *L. lactis* subsp. *lactis* were strong biofilm producers. The remaining strains—two *L. lactis* subsp. *cremoris* and five *L. lactis* subsp. *lactis* strains—were weak producers.

**Figure 3:**
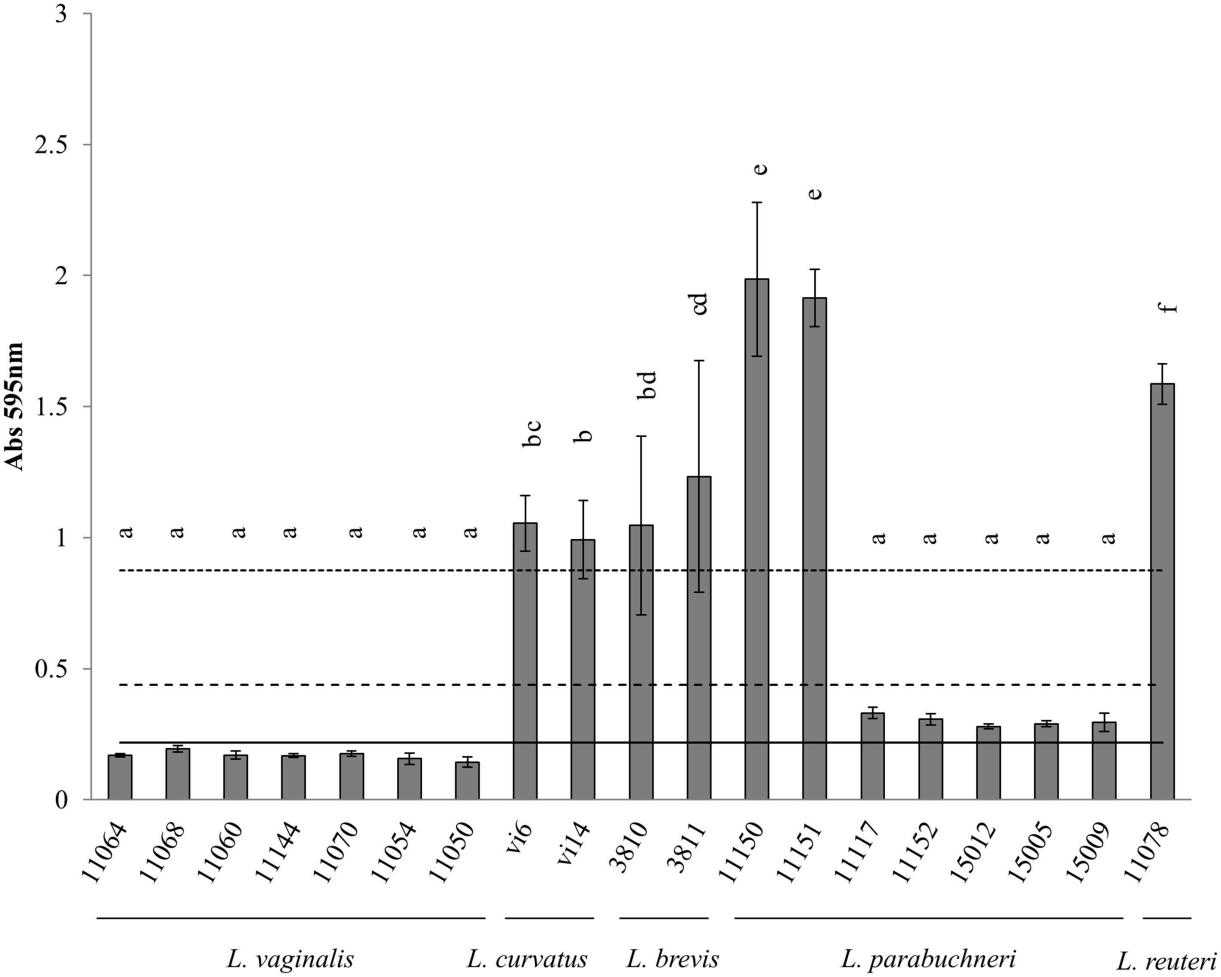
**Biofilm-producing capacity on polystyrene of the biogenic amine-producing *Lactobacillus* strains incubated at 37°C**. The *L. vaginalis, L. curvatus* and *L. brevis* strains were incubated for 24 h. The *L. parabuchneri* and *L. reuteri* strains were incubated for 48 h. Data represent means ± SD (error bars) of three experiments. Values marked with the same letter do not differ significantly (*p* > 0.05 according to the Bonferroni *post-hoc* test). (___) Cut-off line (ODc); (__ __ __) 2 × ODc; (…….) 4 × ODc.

None of the *L. vaginalis* strains were able to form a biofilm (Figure 3). All the *L. curvatus, L. brevis*, and *L. reuteri* strains were, however, strong biofilm producers (Figure 3). Two out of seven *L. parabuchneri* strains were strong biofilm producers, while the remaining strains were only weak producers (Figure 3).

### Bacterial adherence to stainless steel

**Figure 4:**
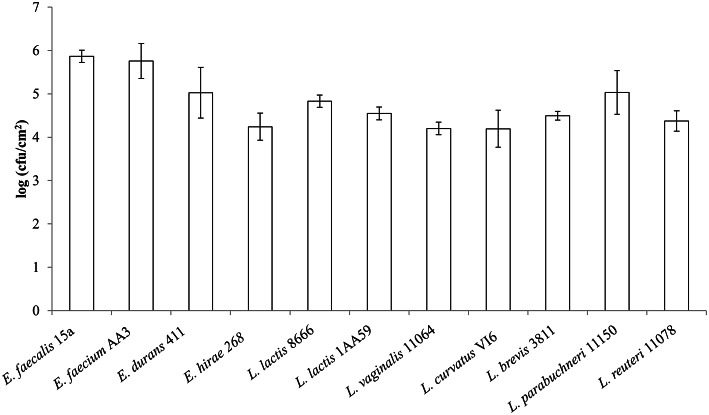
**Adherence to stainless steel coupons by biofilm-producing strains**. Data are expressed as log_10_ cfu/cm^2^ and represent the mean ± SD (error bars) of three experiments.

The strains selected for this assay were the strongest biofilm producers in the polystyrene surface assay, i.e., *E. faecalis* 15a, *E. hirae* 268, *E. durans* 411, *E. faecium* AA3, *L. lactis* subsp. *cremoris* CECT 8666, *L. lactis* subsp. *lactis* 1AA59, *L. curvatus* VI6, *L. brevis* 3811, *L. parabuchneri* IPLA 11150, and *L. reuteri* IPLA 11078. Although, no *L. vaginalis* strain was able to form a biofilm on polystyrene, *L. vaginalis* IPLA 11064 was tested with the steel surface. Two incubation times (24 and 48 h) were tested, but no differences were observed. Figure 4 shows the number of adhered cells of each strain after incubation for 48 h (>10^4^ cfu cm^−2^ for all strains assayed).

### Scanning electron microscopy imaging of cells

SEM photomicrographs of cells adhered to the stainless steel coupons were captured for the same strains as used in the previous assay. Although, no differences were seen in the counts at different incubation times (24 and 48,h), differences in aggregation type and bacterial ultrastructure were observed.

**Figure 5:**
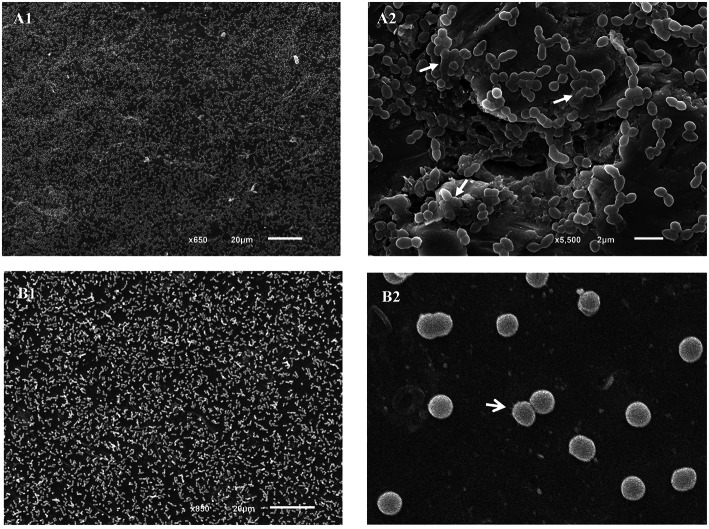
**Scanning electron photomicrographs of *Enterococcus* strains adhered to stainless steel coupons after 24 h of incubation. (A)** *E. faecalis* 15a, **(B)** *E. faecium* AA3. Closed arrows point to the extracellular matrix; open arrows point to anchoring structures.

*E. faecalis* 15a (Figure 5A1), *E. faecium* AA3 (Figure 5B1) and *E. durans* 411 returned clearer images after 24 h of incubation (data not shown) and appeared uniformly spread on the coupons. No adhering *E. hirae* 268 cells were observed at either 24 or 48 h. The *E. faecalis* cells were observed embedded in an extracellular matrix (Figure 5A2); this was not observed for the other two species. Structures that might be involved in anchoring to the surface were observed on *E. faecium* cells (Figure 5B2).

**Figure 6:**
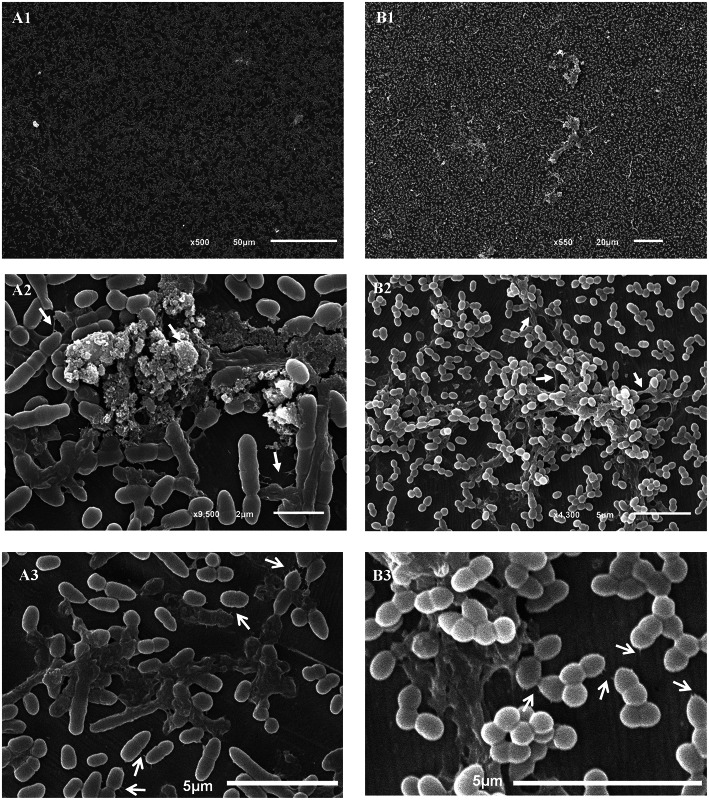
**Scanning electron photomicrographs of *Lactococcus lactis* strains adhered to stainless steel coupons after 24 h of incubation**. **(A)** *L. lactis* subsp *cremoris* CECT8666, **(B)** *L. lactis* subsp. *lactis* 1AA59. Closed arrows point to the extracellular matrix; open arrows point to anchoring structures.

*L. lactis* subsp. *cremoris* CECT 8666 and *L. lactis* subsp. *lactis* 1AA59 returned clearer images after 24 h of incubation. The strain 8666 was uniformly spread across the coupon surface (Figure 6A1), while 1AA59 formed more compact aggregates (Figure 6B1). In both cases, an extracellular matrix was observed, but with a different appearance (see Figures 6A2,A3,B2). Similar structures to those observed in *E. faecium*, and that might be involved in anchoring to the surface, were also observed for both *L. lactis* strains (Figures 6A3,B3).

**Figure 7:**
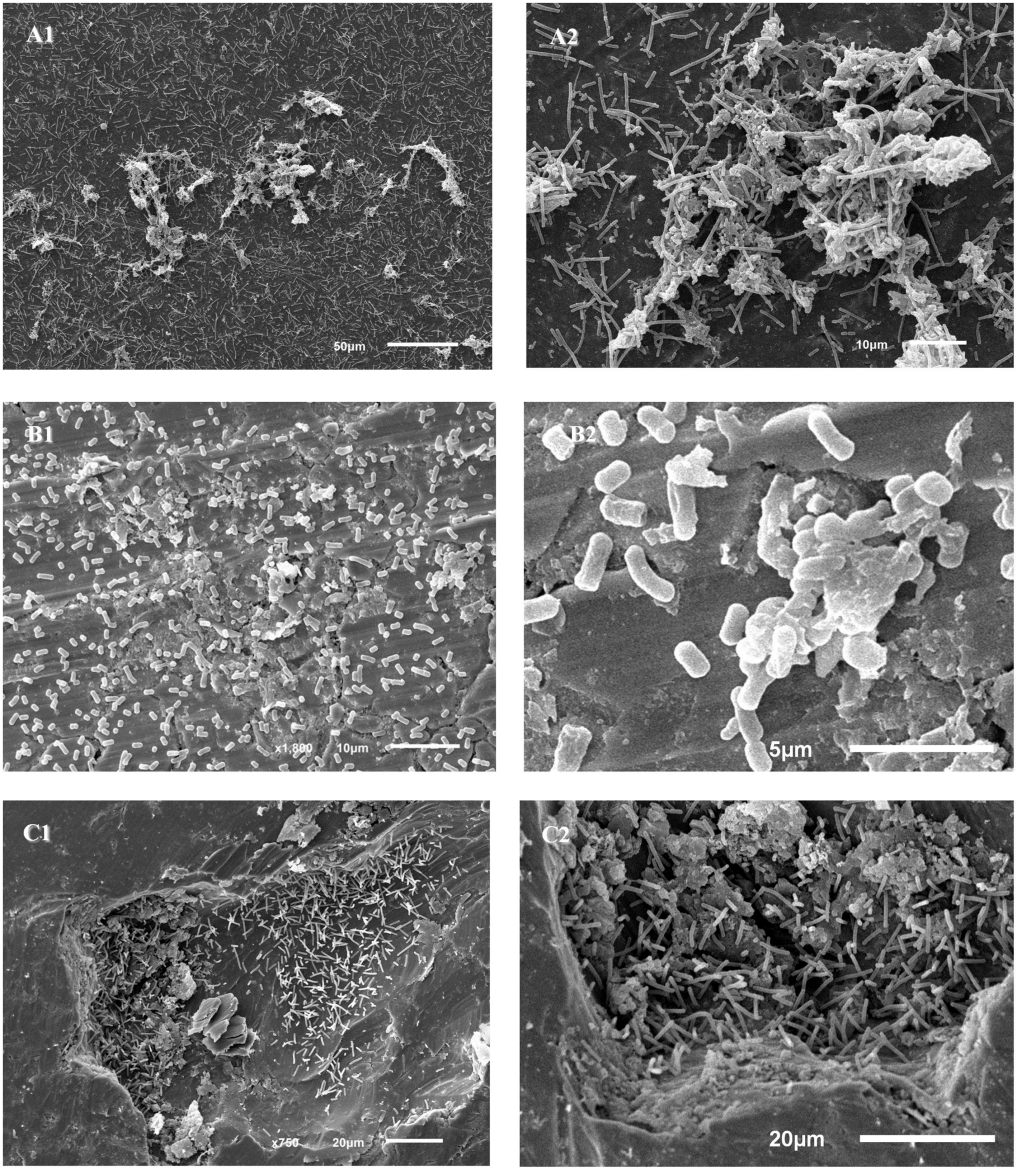
**Scanning electron photomicrographs of *Lactobacillus* strains adhered to stainless steel coupons after 48 h of incubation. (A)** *L. parabuchneri* IPLA 11150, **(B)** *L. reuteri* IPLA 11078, **(C)** *L. brevis* 3811.

*L. parabuchneri* IPLA 11150, *L. reuteri* IPLA 11078, and *L. brevis* 3811 returned clearer images after 48 h of incubation. *L. vaginalis* IPLA 11064 and *L. curvatus* VI6 showed no adhered cells at either 24 or 48 h. *L. parabuchneri* was distributed across the coupon, showing aggregations with an extracellular matrix (Figures 7A1,A2). *L. reuteri* was distributed across the coupons in small aggregations (Figure 7B1) in a clear extracellular matrix (Figure 7B2). *L. brevis* was seen only in the fissures of the coupon (Figures 7C1,C2).

## Discussion

The formation of BA in fermented foods by bacteria, especially cheese, is a cause of much concern. The industrial equipment used in cheesemaking and processing is a source of contamination, and the ability of microorganisms to adhere to the surfaces of such equipment increases their contamination potential. In this work, the ability of 56 BA-producing dairy LAB strains to adhere to a polystyrene surface was examined using the CV assay, a technique that allows easy quantification of total biofilm biomass. The strongest biofilm producers of each species were then examined for their ability to adhere to stainless steel coupons, and the adhered cells observed by SEM.

The formation of biofilms by clinical *Enterococcus* isolates has been much studied since it affects pathogenic potential (Langsrud, 2009). However, enterococci are also a cause of concern for the food industry, and the biofilm-forming ability of several food-related *Enterococcus* species has also been studied (Gomes et al., 2008; Jahan and Holley, 2014; da Silva Fernandes et al., 2015). In the present study, the biofilm-forming capacity of tyramine- or tyramine and putrescine-producing *Enterococcus* species was examined. The results of the CV assay showed that all the strains analyzed, except for two *E. faecium* strains, were able to form biofilms, although the total biomass produced differed. These results agree with those of previous studies that showed several foodborne *E. faecalis* and *E. faecium* isolates to be either weak, moderate or strong biofilm producers, while some isolates formed no biofilm at all (Gomes et al., 2008; Jahan and Holley, 2014). *E. durans* has also been described as a strong or moderate biofilm producer (Amel et al., 2015; Pieniz et al., 2015). The only strain of *E. hirae* analyzed in the present work was a weak biofilm producer; to our knowledge, the capacity of this species to form biofilms has not been previously studied.

All the *Enterococcus* strains selected to see whether they could adhere to stainless steel did so, with counts reaching 10^4^ cfu cm^−2^. The capacity of *E. faecalis, E. faecium*, and *E. durans* to adhere to stainless steel has been previously reported (Amel et al., 2015; da Silva Fernandes et al., 2015). The *Enterococcus* strains that adhered to the stainless steel coupons were observed by SEM, except for *E. hirae* 268; - this strain attached only weakly to the metal surface and was unable to resist the treatment required prior to observation. Of the strains that covered the surface of the steel coupons (Figure 5), none formed the complex three-dimensional structures reported by da Silva Fernandes et al. (2015). Some aggregations of *E. faecalis* 15a cells were observed (Figure 5A2), while *E. faecium* AA3 cells appeared separated from one another, but all cells were sufficiently well adhered not to be detached by the PBS washes or the treatment required prior to SEM observations. To our knowledge, this is the first time that images of *E. durans* cells adhered to stainless steel have been captured.

The ability of *L. lactis* to form a biofilm on a surface has been related to the latter's physicochemical properties (Giaouris et al., 2009; Oxaran et al., 2012). However, few studies have ever been performed on *L. lactis* biofilm formation. In the present work, 12 putrescine-producing *L. lactis* strains were shown by the CV assay to be either weak or strong biofilm producers. The two strains that most strongly formed biofim on polystyrene—*L. lactis* subsp. *cremoris* CECT 8666 and *L. lactis* subsp. *lactis* 1AA59—also adhered to stainless steel, reaching counts of 10^4^ cfu cm^−2^. SEM images showed cells of both strains to be embedded in an extracellular matrix and to be clearly adhered to the coupons. This is the first time that *L. lactis* adhered on stainless steel have been observed by SEM. Unlike that seen for enterococci, the *L. lactis* CECT 8666 cells showed tridimensional structures including filamentous ones that might help them adhere to the surface (Figures 6A2,A3). The extracellular material formed a pod-like covering over the cells (Figures 6A2,A3). *L. lactis* 1AA59 cell clusters also appeared to be attached via the extracellular matrix (Figure 6B2). For both *L. lactis* strains (Figures 6A3,B3) examined, and *E. faecium* AA3 (Figure 5B2), small protuberances of the cell surface were observed, which may have helped anchor the cells to the coupons. Certainly, aggregation and biofilm formation in *L. lactis* has been associated with the production of functional pili (Oxaran et al., 2012). Although, the adhesion of the putrescine-producing strains analyzed in this work would be undesirable for the food industry, allowing the development of *L. lactis* biofilms has been proposed as a means of preventing the growth of pathogens on food industry surfaces (Leriche et al., 1999; Zhao et al., 2004).

The presence of several Lactobacillus species (*L. curvatus, Lactobacillus fermentum, Lactobacillus delbruekii, Lactobacillus paracasei, Lactobacillus plantarum*, and *L. reuteri*) on the surfaces of dairy equipment has been reported (Somers et al., 2001; Scatassa et al., 2015). In the present work, two tyramine and putrescine-producing *L. brevis* strains were strong biofilm producers on polystyrene. This agrees with previous results for *L. brevis* strains isolated from onions (Kubota et al., 2008). The two tyramine-producing *L. curvatus* strains tested were also strong biofilm producers. Biofilm formation by *L. curvatus* has been previously described, although it was found to be a weaker biofilm producer than in the present work (Pérez Ibarreche et al., 2014). Fifteen histamine-producing strains, seven belonging to *L. vaginalis*, seven to *L. parabuchneri* and one to *L. reuteri*, were examined by the CV assay. The seven histamine-producing *L. vaginalis* strains were unable to form biofilms. All the *L. parabuchneri* strains were able to form biofilms, and were either weak or strong biofilm producers, depending on the strain. This agrees with the results of previous reports (Diaz et al., 2016b). The *L. reuteri* strain examined was a strong biofilm producer. Numerous studies have described the formation of biofilms by *L. reuteri*, some strains of which are considered probiotics (Leccese Terraf et al., 2014). A system regulating biofilm formation in *L. reuteri* was recently characterized (Su and Ganzle, 2014). In the present work, *L. reuteri* IPLA 11078, along with *L. vaginalis* IPLA 11064, *L. curvatus* VI6, *L. brevis* 3811 and *L. parabuchneri* IPLA 11150 were all able to adhere to the stainless steel coupons, and reached counts of over 10^4^ cfu cm^−2^. However, when the coupons were observed under the SEM, only *L. parabuchneri* IPLA 11150 (Figure 7A) and *L. reuteri* IPLA 11078 (Figure 7B) cells were seen attached. For *L. parabuchneri* IPLA 11150, the cells aggregated into clumps composed of long chains of undivided cells. A previous study on *Pseudomonas aeruginosa* showed that elongated cells are inclined to form cohesive clumps (Yoon et al., 2011). According to the CV assay, the present *L. parabuchneri* and *L. reuteri* strains were strong biofilm producers. However, while the same assay suggested *L. curvatus* VI6 and *L. brevis* 3811 to be strong biofilm producers, the absorbance measured was very close to the lower limit for such classification. The few cells seen attached to the coupons might be the result of them not being able to resist the treatment required prior to SEM observation. This would appear to be supported by the fact that *L. brevis* 3811 was not homogenously attached to the surface of the coupons, but seen in their fissures. This is an important finding for the food industry since steel surfaces do develop cracks and these appear able to protect bacteria from cleaning procedures.

The present results show all the species examined, except *L. vaginalis*, had BA-producing strains able to form biofilms and that they could adhere to stainless steel, a material commonly used to make equipment in the food industry. Although, the cells attached to the stainless steel coupons commonly showed none of the three-dimensional structures reported by da Silva Fernandes et al. (2015), counts of over 10^4^ cfu cm^−2^ were recorded in all cases, with *E. faecalis, E. faecium, E. durans*, and *L. parabuchneri* exceeding 10^5^ cfu cm^−2^. Given the large surface area of industrial equipment, BA-producing bacteria that can adhere to steel clearly pose a food contamination threat. This problem is of particular concern in the dairy industry since post-ripening treatments such as cheese grating bring food into close and prolonged contact with equipment surfaces. In fact, it has already been shown that cheese that has undergone post-ripening processing (cutting, slicing, or grating) has higher histamine levels than non-processed cheese (Ladero et al., 2009). It is therefore important that our knowledge of the adhesion and biofilm forming capacities of BA-producers be improved, to prevent food contamination by these spoilage bacteria and eventually the accumulation of biogenic amines in food.

## Author contributions

MD carried out the experiments and drafted the manuscript; BR, VL collaborated in conducting some experiments; VL, Bd, and MF participated in the study design and writing of the manuscript; MC, MA provided the general concept, designed the experiments, and supervised the experimental work and the manuscript. All authors contributed to the discussion of the research and approved the final manuscript.

### Conflict of interest statement

The authors declare that the research was conducted in the absence of any commercial or financial relationships that could be construed as a potential conflict of interest.
